# Case Report: Role of an Iodinated Rectal Hydrogel Spacer, SpaceOAR Vue™, in the Context of Low-Dose-Rate Prostate Brachytherapy, for Enhanced Post-Operative Contouring to Aid in Accurate Implant Evaluation and Dosimetry

**DOI:** 10.3389/fonc.2021.810955

**Published:** 2021-12-22

**Authors:** Andrew Gross, Jiankui Yuan, Daniel Spratt, Elisha Fredman

**Affiliations:** Department of Radiation Oncology, Seidman Cancer Center, University Hospitals Cleveland Medical Center, Cleveland, OH, United States

**Keywords:** prostate cancer, brachytherapy, radiation, SpaceOAR Vue, toxicity

## Abstract

We present a case series of 13 consecutive patients with prostate cancer treated with low-dose-rate (LDR) brachytherapy, utilizing SpaceOAR Vue™, the recent iodinated iteration of the SpaceOAR™ hydrogel rectal spacer. Low- and favorable intermediate-risk patients receiving monotherapy and unfavorable intermediate- and high-risk patients undergoing a brachytherapy boost were included. Permanent brachytherapy can result in subacute and late rectal toxicity, and precise contouring of the anterior rectal wall and posterior aspect of the prostate is essential for accurate dosimetry to confirm a safe implant. Clearly visible on non-contrast CT imaging, SpaceOAR Vue™ can substantially aid in post-implant contouring and analysis. Not previously described in the literature in the context of LDR brachytherapy, we demonstrate the added clinical benefit of placing a well-visualized rectal spacer.

## Background

Low-dose-rate (LDR) brachytherapy with permanent implantation of radioactive seeds is a highly effective method of delivering curative radiation therapy for prostate cancer (1, 2). As monotherapy for low- and favorable intermediate-risk disease and in combination with external beam radiation (EBRT) for unfavorable intermediate- and high-risk disease, LDR brachytherapy yields biochemical progression-free survival rates of approximately 96 and 89–94%, respectively (3–5). In fact, compared to all other radiation therapy modalities, LDR brachytherapy allows for the highest possible dose to be delivered to the prostate gland (6). It is this very feature, however, that makes limiting exposure to surrounding critical structures, as assessed by precise contouring and dosimetric calculation, particularly important.

Anatomically, the rectum is situated immediately posterior to the posterior aspect of the prostate and is typically the dose-limiting adjacent tissue. While this structure can tolerate a certain degree of radiation exposure, an excessive dose to a given point or a volumetric region can result in subacute and late side effects that range from bothersome to potentially life-threatening (7–10). Therefore, when calculating radiation dose to the anterior rectal wall as part of the post-operative implant evaluation, accurate demarcation and contouring of the rectum and its position relative to the posterior aspect of the prostate is essential (11). This is especially important in LDR brachytherapy due to the exceptionally high radiation doses delivered and the permanent nature of the seeds, yet it is uniquely challenging due to a degree of local bleeding and edema caused by the transperineal brachytherapy procedure and CT artifact from the metallic seeds or other high-density materials.

One method to decrease radiation dose to the rectum, to reduce the risk of rectal toxicity, is transperineal injection of a biomaterial into the potential fat space between Denonvilliers’ fascia and the anterior rectal wall (12). Polyethylene glycol (PEG)-based absorbable hydrogel SpaceOAR™ (Boston Scientific Corporation) reduces both objective high-dose exposure to the rectum and patient-reported rectal toxicity in the setting of EBRT and stereotactic body radiotherapy (12–14). This FDA-approved rectal spacer is now widely used when delivering high-dose prostate radiation therapy.

**Figure 1:**
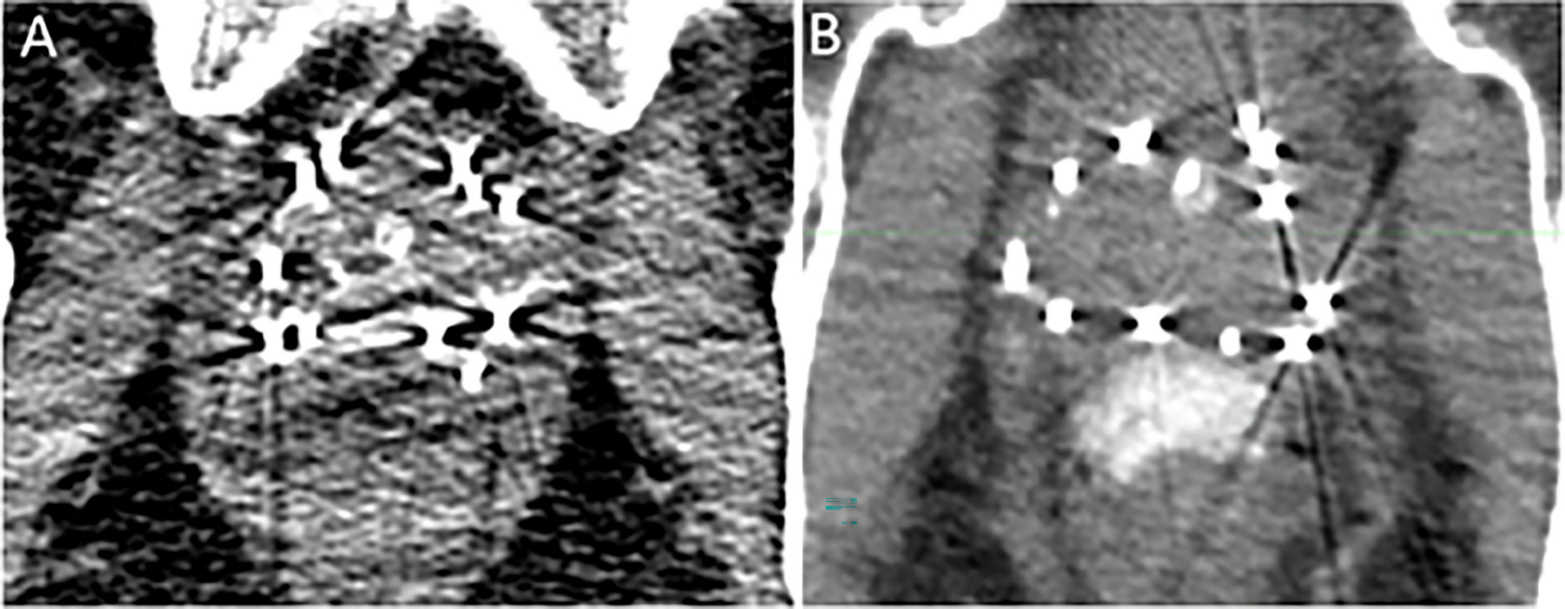
Day 0 non-contrast CT scan for postoperative dosimetry following low-dose-rate brachytherapy with placement of a standard SpaceOAR™ **(A)** and SpaceOAR Vue™ **(B)**.

PEG hydrogel application in the context of LDR brachytherapy presents a unique challenge for post-operative dosimetry (15). While hyperintense on T2-weighted MRI, the hydrogel appears isodense on CT, and even though external radiation is frequently planned utilizing MRI fusion, post-implant dosimetry is most often calculated on CT imaging alone. Following the brachytherapy procedure, the presence of low/moderate-grade edema and bleeding proximal and posterior to the prostate can make it difficult to identify the precise location of the hydrogel and the anterior rectal wall, making proper contouring and accurate post-implant dosimetry a challenge (**Figure 1A**). Furthermore, the streak artifact caused by the brachytherapy seeds as well as by other high-density implanted materials, such as a metallic hip replacement, can obscure the boundaries of the rectal wall.

More recently, a novel iteration of the SpaceOAR™ product was developed, SpaceOAR Vue™, an iodinated cross-linked PEG hydrogel, making the similarly resorbable hydrogel radiopaque easily seen on non-contrast CT. The properties of the standard SpaceOAR™, namely, transformation from liquid components to a solid gel-like material in fractions of a second, stability over at least 3 months, and being hydrolyzed and renally cleared at approximately 6 months, are unchanged in the iodinated version (16). The primary function of the SpaceOAR Vue™, namely, stable and consistent peri-rectal spacing, has recently been objectively compared to that of its non-iodinated predecessor. In a rigorous longitudinal comparison analysis, it was found that, despite a moderately overall smaller average delineated volume, SpaceOAR Vue™ provided comparable relative prostate–rectal separation, appropriate positioning, stability over a course of EBRT, and dosimetric consistency without significant changes in plan quality or robustness relative to SpaceOAR™ (17).

Specifically, its distinct visibility on non-contrast CT potentially makes it particularly beneficial as a rectal spacer in the setting of LDR brachytherapy, providing the same benefits of anterior rectal wall protection but with improved visibility to facilitate correct contouring of the posterior prostate and rectal wall after the implant. To date, there exists minimal literature detailing the advantages of the SpaceOAR Vue™, and to the best of our knowledge, there are no prior reports of SpaceOAR Vue™ placement in the setting of LDR brachytherapy. Here we present a case series of 13 sequential patients with localized prostate cancer treated with LDR brachytherapy, either as monotherapy or as a boost in conjunction with EBRT, who underwent placement of SpaceOAR Vue™ at the time of implant.

### Case Series Presentation

Thirteen male patients received definitive treatment for localized prostate cancer between November 2020 and July 2021 (**Table 1**). No patient had a history of an inflammatory bowel disease, other baseline auto-immune diagnoses, nor past abdominal or pelvic surgeries. Pathologic diagnoses were made based on 12-core transrectal ultrasound (TRUS)-guided biopsy, and the patients underwent diagnostic multiparametric MRI (mpMRI). All patients had clinical stage T1c-2c disease and Gleason scores 6–8 and were stage groups I and IIC. Pre-treatment PSA ranged between 4.78 and 10.27 ng/ml. A total of 54% of the patients had low- or favorable intermediate-risk disease and were treated with LDR monotherapy, and 46% had unfavorable intermediate- or high-risk disease and received brachytherapy as part of a boost at 1 to 2 weeks following EBRT (18). EBRT for those undergoing combination therapy was delivered to the prostate and seminal vesicles, with or without coverage of the pelvic lymph nodes depending on the calculated risk of involvement, prescribed up to 45 Gy in 1.8-Gy daily fractions using volumetric modulated arc therapy and daily cone beam CT. Androgen deprivation therapy, as indicated per national standards, was initiated approximately 2 months prior to EBRT.

**Table 1:** Patient characteristics.

Age (years): mean (range)	65.5 (59–73)
Gleason Score	
6	3
7	9
8	1
PSA (ng/ml): mean (range)	7.0 (4.78–10.27)
Stage group	
I	3
IIB	6
IIC	4
Monotherapy	7
Boost	6
ADT	6
Gland volume (cc): mean (range)	35.2 (26.6–53.1)

Brachytherapy preplanning was performed utilizing mpMRI (19). The mean prostate volume was 35.2 ± 8.1 cc (range, 26.6–53.1cc). Any radiographically identified high-grade (PIRADS 4 or 5) lesion (20) which corresponded to a positive region on diagnostic biopsy was contoured and planned for dose escalation of 150–200% of the prescription dose. SpaceOAR Vue™ was placed at the time of the brachytherapy implant (**Figure 1B**).

**Figure 2:**
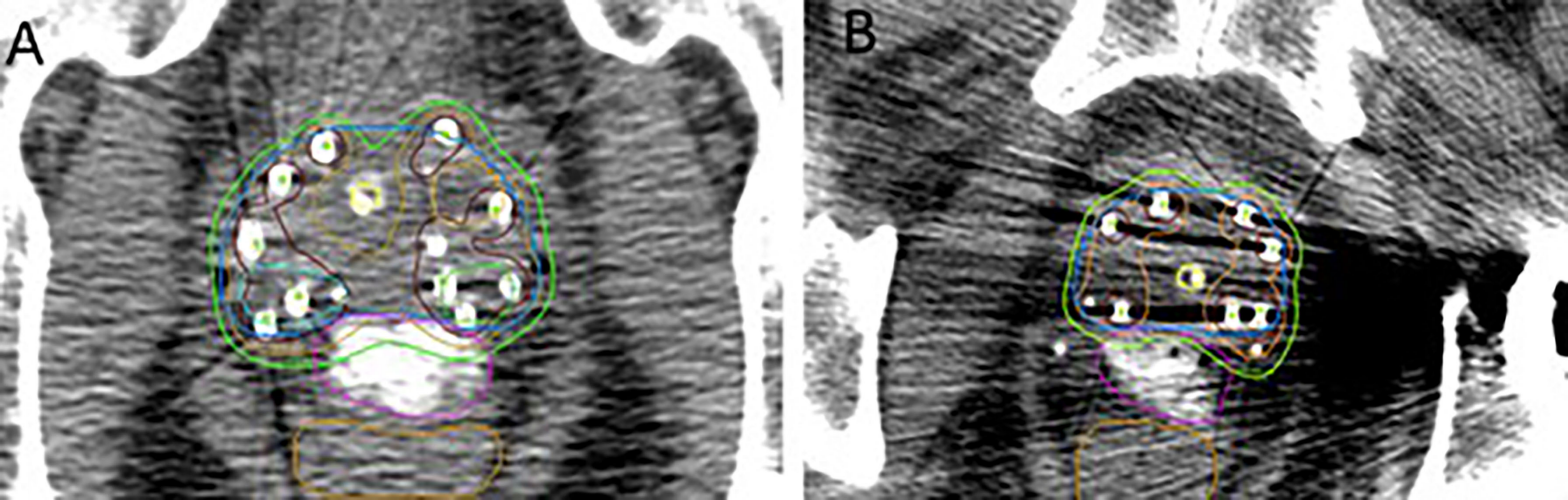
**(A)** Bilateral posterior margin high-grade PIRADS lesion dose escalated to 200% of the prescription dose. **(B)** Patient with metallic left total hip replacement. Contours: blue, prostate gland; pink, SpaceOAR Vue™; brown, rectum; green, 100% isodose line; orange, 150% isodose line; maroon, 200% isodose line; aqua and light green, PIRADS lesions.

Among the patients treated, two were notable for clinical presentations in which SpaceOAR Vue™ had the potential to offer particular benefits. One was found to have two high-grade lesions, bilaterally concordant on mpMRI and biopsy, at the posterior boundary of the prostate at mid-gland (**Figure 2A**). A second patient had a metallic total hip replacement which generated substantial imaging artifact in the region of the prostate and rectal wall interface (**Figure 2B**).

## Methods

The implants were performed in an operating room setting under general anesthesia. Pre-operative weight-based antibiotics were given prophylactically. The patients were placed in the dorsal lithotomy position and prepped and draped in the usual sterile fashion. Using TRUS, multi-planar imaging of the prostate was obtained and captured in 5-mm increments from the base to the apex. These images were then intraoperatively fused to the preplanned T2-weighted MRI, and the MRI-based preplan was adjusted to account for observed variations in patient positioning. Employing this intraoperative plan as well as real-time planning with adaptive virtual dosimetry, I-125 radioactive seeds were transperineally placed under direct visualization with a modified peripheral loading technique. Definitive and boost cases were treated to doses of 145 and 110 Gy, respectively, prescribed to the periphery of the prostate gland per the current American Brachytherapy Society recommendations (18). MRI-visible high-grade PIRADS lesions were boosted up to 150–200% of the prescription dose. The mean number of needles and seeds required was 18.6 (range, 15–26) and 62.2 (range, 45–95), respectively.

After insertion of the final brachytherapy seed, the perineal grid was removed and the SpaceOAR Vue™ hydrogel was assembled. For patients who received an initial 45 Gy of EBRT, the ability to successfully hydrodissect the perirectal space was first confirmed using a 6-in. 18G needle and sterile saline prior to opening the hydrogel kit. SpaceOAR Vue™ placement was performed as previously described (12). The provided 18G needle was advanced under TRUS guidance, and the appropriate placement of the needle tip in the perirectal fat at midgland was confirmed in both axial and sagittal planes. Hydrodissection was performed to identify and open the potential space. The SpaceOAR Vue™ Y-connecter was then attached to the 18G needle, and the hydrogel components were deployed over approximately 10–12 s, taking into account the more viscous nature compared to the standard SpaceOAR™. The mean separation between the prostate and the rectum was 12.2 ± 2.1 mm (8.6–16.3mm), with a mean width of 29.0 mm (19.8–40.2mm) and length of 47.2 mm (34.3–64.9 mm).

Upon recovering from anesthesia, the patients underwent a same-day non-contrast CT (foley in place) for immediate post-operative dosimetry. All patients were discharged to home on the same day after independently voiding and were prescribed five additional days of prophylactic oral antibiotics as well as 0.8 mg of an α_1_ adrenergic antagonist (tamsulosin) to ease post-brachytherapy irritative urinary symptoms. Contouring of the prostate, urethra, rectum, and SpaceOAR Vue™ was performed in collaboration by the treating physician and the physicist. A favorable dose coverage of the prostate was achieved in all cases, with a mean prostate D_90_ of 113.8 ± 7.5% (range, 101.4–125.3%) and V_100_, V_150_, and V_200_ of 96 ± 2.4, 59.1 ± 11.5, and 31.7 ± 8.1%, respectively. Excellent rectal sparing was calculated, with a mean rectal D0.1cc, D1cc, and D2cc of 70.6, 49.6, and 41.8 of the prescribed dose, respectively.

No immediate procedure-related complications were reported within the week following brachytherapy. Toxicities were formally assessed at 1- and 3-month time periods. At 1-month follow-up, 54% of the patients experienced grade 2 urinary toxicity, and 46% had grade 0–1 urinary toxicity, comprised primarily of urgency and frequency, as expected. Relative to assessment at the time of presentation, there was a reported mean increase of 4.3 points on the International Prostate Symptom Score (IPSS) screening tool. By 3 months, only 38.5% maintained persistent grade 2 urinary toxicity, and the patients reported a mean decrease of 4 points in IPSS compared to baseline. At 1-month follow-up, 92% reported no rectal toxicities, with only one patient experiencing grade 1 mild diarrhea. The patients reported no rectal toxicities at 3 months post-brachytherapy.

## Discussion

The dosimetric and clinical benefits of a rectal spacing hydrogel during radiation therapy in the treatment of prostate cancer are well documented (12–14). To the best of our knowledge, this is the first report in the literature on the usage and advantages of iodinated SpaceOAR Vue™ in the setting of LDR brachytherapy for prostate cancer. In addition to the known benefit of displacing the anterior rectal wall away from the prostate and the high-dose region of radiation achieved with SpaceOAR™, the clear visibility of SpaceOAR Vue™ on CT makes it particularly beneficial for correctly identifying the precise boundaries of the posterior aspect of the prostate and anterior rectal wall in the unique context of post-implant dosimetry. Unlike with external radiation therapy, this anatomic region contains substantial CT artifact due to post-operative localized bleeding and edema as well as from the implanted metal seeds. We demonstrate even further benefit for patients who have a high-density hip implant, the effect of which can greatly obscure the anatomy of interest on CT.

Prior to recommending the addition of any invasive procedure to reduce potential risk, consideration must be given to the possible risks of the procedure itself as well as to the true extent of the purported benefit. While the overall risks of SpaceOAR™ insertion appear quite low, there have been documented reports of catastrophic complications, and because of that combined with remaining questions regarding the extent of the objective advantages across all patients treated for prostate cancer, responsible equipoise is appropriate in deciding to whom the device should be recommended (21). The specific population of patients in this report, wherein an invasive procedure was performed to implant a permanent dose far above that given with EBRT in close proximity to the adjacent rectum, perhaps represents one of the particular groups where the benefits of SpaceOAR™, and specifically SpaceOAR Vue™, emerge.

The risk of rectal toxicity from LDR brachytherapy is directly related to high-dose radiation exposure to sub-volumes of the rectum which is associated with the distance of the rectum to the brachytherapy seeds. The perirectal spacing and rectal dosimetry achieved in our series compare favorably with those of other recent reports of patients undergoing LDR brachytherapy with SpaceOAR™. In their retrospective series, Taggar et al. reported a mean separation of 11.2 mm, yielding a D1cc of the rectum at 25.3% in the context of good target coverage (prostate V_100_ and D_90_ of 94.0 and 112.4%, respectively (22), results which were comparable to those in our experience.

Kahn et al. reported rectal toxicities in patients receiving LDR brachytherapy with or without a PEG hydrogel rectal spacer (23). Patients with hydrogel placement had fewer acute grade 1 rectal toxicities reported at 1-month follow-up compared with those without hydrogel (12.5 *vs.* 17.5%). Among the 40 patients who did not have a hydrogel placed, one experienced delayed grade 2 rectal toxicity. The overall rates of grade 2+ rectal toxicity following LDR brachytherapy are low, ranging from 2.2 to 26% (weighted average of 7.9%) (24–35). The reporting rates in aggregate is complicated by differing toxicity criteria used and variations in toxicity time points between studies. Furthermore, grade 1 toxicity is not commonly included but can contribute to decrements in the quality of life (36). In our series of SpaceOAR Vue™ placement, only one patient reported grade 1 rectal toxicity at 1 month, and none was reported at 3 months.

**Figure 3:**
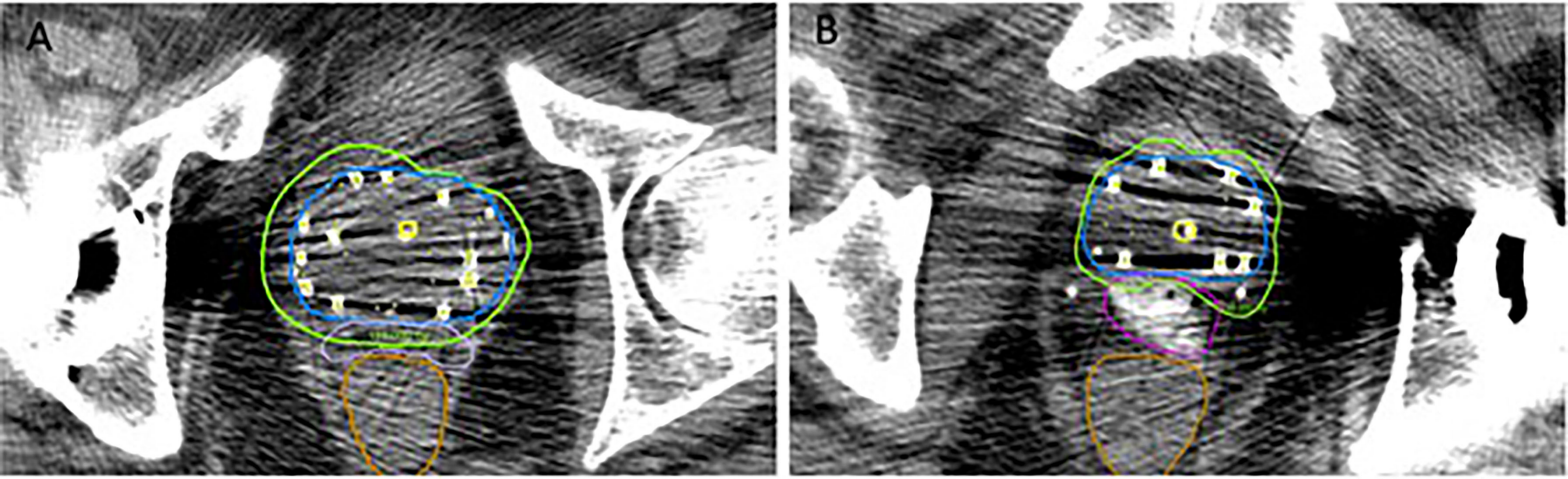
Comparison between SpaceOAR™ **(A)** and SpaceOAR Vue™ **(B)** in cases with artifact from total hip replacement.

Certain clinical scenarios may especially benefit from the clearly visualized iodinated SpaceOAR Vue™. In addition to the hardening and streak artifact generated by the seeds themselves, the pelvic CT images of patients who have undergone metallic hip replacement surgery often show additional substantial artifact that can obscure the prostatic/rectal interface (**Figures 2B**, **3**) (37). SpaceOAR Vue™, in this context, is uniquely capable of improving the ability to discern the precise boundary of the posterior aspect of the prostate and anterior rectal wall (**Figure 3**). A second scenario is when attempting to dose-escalate or boost a known region of high-grade disease (**Figure 2A**). This is an emerging technique which has been reported to improve the biochemical progression-free survival. In a series of prospective randomized controlled trials, FLAME and hypo-FLAME demonstrated feasibility, improved biochemical outcomes, and limited increases in acute toxicity with focal dose escalation of MRI-visible high-grade lesions (38–40). Safely delivering such elevated doses even further requires accurate contouring and plan evaluation with regard to organs at risk. When delivering LDR brachytherapy, we typically boost mpMRI high-grade lesions concordant with biopsy results up to 150–200% of the prescription. Within this cohort, we highlight a particular case of bilateral posterior lesions in the prostate that were treated with this degree of dose escalation. SpaceOAR Vue™ allowed for improved accuracy in delineating the anterior rectal wall to assure an appropriate brachytherapy implant.

### Limitations

The limitations of this review include its inherent nature of being a retrospective case series and its lack of controlled comparison. Although an observational report, all 13 patients were treated by the same attending physician in collaboration with the same physicist, both with extensive experience in LDR brachytherapy, thereby reducing inter-patient planning, treatment, and contouring variability. A second limitation is the timeframe in which post-operative dosimetry was performed. Per the American Brachytherapy Society guidelines, post-operative dosimetry should be performed between days 0 and 60 from the procedure (18). The benefits of day 0 dosimetry include convenience, immediate information on the success of the implant, and enhancement of trainee education, while later dosimetry (days 30–60) allows for the resolution of edema and more accurately reflects the true deposited dose. As per department protocol, post-implant dosimetry was performed on day 0 for patients in this cohort. As such, there was more edema present during post-plan contouring and evaluation than would have been detected later. This schedule, however, is commonly followed in many practices and reflects a real-world clinical scenario in which SpaceOAR Vue™ can offer benefits.

## Conclusion

SpaceOAR Vue™ offers additional benefits compared to the prior iteration of the hydrogel rectal spacer which is particularly advantageous in the unique context of LDR brachytherapy. It is not difficult to use, and like its predecessor, it results in significantly decreased high-dose radiation exposure to the rectal wall. Specifically, in the context of LDR brachytherapy where accurate post-op dosimetry is critical, the improved ability to identify the posterior aspect of the prostate and anterior rectal wall can offer improved radiation planning and therefore overall patient care. As such, we recommend the use of SpaceOAR Vue ™ in place of the standard SpaceOAR™ in cases of LDR brachytherapy utilized to treat patients with prostate cancer.

## Data Availability Statement

The raw data supporting the conclusions of this article will be made available by the authors, without undue reservation.

## Ethics Statement

The study involving human participants was reviewed and approved by the Internal Review Board of University Hospitals. The patients/participants provided their written informed consent to participate in this study. Written informed consent was obtained from the individual(s) for the publication of any potentially identifiable images or data included in this article.

## Author Contributions

AG contributed to the writing of the manuscript and authored the initial draft. JY was the radiation physicist who assisted with all of the brachytherapy procedures, pre- and post-planning, was involved with creating figures as well as reviewed the manuscript. DS aided with the review of the manuscript. EF was the principal investigator who developed the concept for the article, performed the brachytherapy procedures, including reviewing and approving all pre- and post-plans, and drafted and revised the manuscript. All authors contributed to the article and approved the submitted version.

## Funding

The publication of this manuscript was supported by Seidman Cancer Center, University Hospitals Cleveland Medical Center.

## Conflict of Interest

EF is a paid speaker and consultant for Boston Scientific.

The remaining authors declare that the research was conducted in the absence of any commercial or financial relationships that could be construed as a potential conflict of interest.

## Publisher’s Note

All claims expressed in this article are solely those of the authors and do not necessarily represent those of their affiliated organizations, or those of the publisher, the editors and the reviewers. Any product that may be evaluated in this article, or claim that may be made by its manufacturer, is not guaranteed or endorsed by the publisher.
